# Bibliographical Notices

**Published:** 1856-06

**Authors:** 


### BIBLIOGRAPHICAL NOTICES.

*The Principles and Practice of Ophthalmic Medicine and Surgery.* By T. WHARTON JONES, F. R. S., Professor of Opthalmic Medicine and Surgery in University College, London; Opthalmic Surgeon to the Hospital, &c. *With one hundred and ten illustrations. Second American edition, with additions, from the second and revised London edition.* Philadelphia: Blanchard and Lea, 1856. 12mo., pp. 500, including glossary and index.

This is an old and well known occupant of the office and the student's table. Although not inferior, considering the nature of its subject, to any of its companions of the Churchill series, in the scope and character of its contents, it is, we believe, the only one of the American reprints of that series which has been allowed to appear in its original duodecimo form. It may therefore be regarded as fulfilling the requisites of a manual more nearly than most of its fellows from the same American source; and we are disposed, on that account especially, to recommend it, and to hope that in these days of unwieldy volumes it may receive a hearty welcome from the crowd of students and practitioners, whom it might save from the bewilderment of greater books, if not the more questionable bliss of entire ignorance of their subject. We do not mean seriously to advocate a manual in preference to the systematic treatise, above all such as we already have attainable on diseases of the eye; we desire only to call attention to Mr. Wharton Jones' work as a desirable specimen of its class. Without aiming to be more than "a text-book for students and a book of reference for practitioners," it is sufficiently comprehensive and clear, as well as generally exact and reliable in its exposition of the principles and practice of ophthalmology. It thus offers to students and practitioners of limited means and opportunities—to all, in short, who look to hand-books for their guidance—an amount of information in relation to a difficult yet unavoidable branch of practice, which they will hardly find elsewhere in a shape more available or convenient for their particular use.

The new edition appears to be a decided improvement on its predecessor in more than one respect. The additions of the author in text and illustrations, have brought his work sufficiently up to the date of publication without material effect upon its former size; while the alterations of style and arrangement observable throughout the volume, have very much improved the appearance of the text and general attractiveness of the contents, at the same time that, in the sections and chapters, the old order is preserved.

The labors of the American Editor have been confined to the revision of the English copy for the press,—a matter of considerable moment in pages crowded as these are with numerical references, Greek derivatives and other complicated technicalities—all of which appear to have been attended to with commendable fidelity, and to the introduction of a few "short notes in relation to details of treatment, in which the experience of this city appeared to him to differ from that of London."

We are thankful that his disinclination to increase the bulk of the volume, or to interfere with most portions of the English text, has led him to confine himself entirely to matters of practical detail, and to aim at rareness as well as brevity in his interpolations. Our views in regard to the editing of foreign publications, and especially to the practice of introducing "American Notes" into unauthorized editions of other men's works, have already been expressed. It is becoming every day more evident that they are in accordance with the prevailing sentiment of the profession. We believe that they are, now at least, shared in by the best known and most active among those who have been singled out to undertake these thankless offices, and by none more justly, perhaps, than the editors of the manuals of which the one before us is the latest. Few of them, we suspect, are still hugging the delusion that their reputations are benefitted, or their time and labor requited, in any way, through such equivocal connections; and more than one, we doubt not, would gladly escape the memory of sundry enduring instances of former "zeal and industry" thus miserably wasted.

There does, however, seem to be some excuse in the idea of adding notes respecting practical details of treatment, which extended experience has satisfied the editor to be necessary in order to render the precepts of the book endorsed by him safe and practicable among those who are asked to be guided by it. It is one of the misfortunes of an editor of a reprint—one of the miseries of the situation, that he must be liable to dilemmas of this kind—that if he undertakes to play the part of Sinbad in any shape, a part which the profession seems generally to think had better not be undertaken at all unless there are palpable reasons for so doing, he must assume and propagate his author's errors, or, by attempting to correct them with caveats and qualifications, expose his unlucky head to the tender mercies of all the critics and cavillers, whether *know nothings* or not, who may choose to spend their words upon him.

We know enough of Wills' Hospital for Diseases of the Eye, and of its Surgeons, to be satisfied that the latter are fully competent to determine upon the proper course of treatment, and the best mode of operating for any of the forms of disease which are presented in its wards; nor are we disposed to deny them the right of modifying treatment and operations which have been recommended in other countries, so far as to adapt them to the requirements of their experience on patients subjected to their observation here. This is all that we understand our editor, from his preface, to have attempted, and this he has done only to a limited degree.

A reference to the notes will show the reader that they are not numerous, in most cases very brief, always concise and invariably practical. Under these circumstances we confess ourselves but little inclined to complain of their intrusion, especially as Mr. Jones' directions are not such as our own experience would warrant us in following in many places which have been noted and, we think, corrected by Dr. Hartshorne. Indeed, the therapeutic details, under the different heads, although in the main judicious and precise, and, perhaps, full enough for careful and competent prescribers, struck us as the most defective portion of the book—certainly unequal in merit and completeness to the physiological and pathological and most of the operative sections; and we are not surprised that the editor has yielded to the temptation to fill up the few gaps which he has ventured to occupy.

The two longest notes in the book are devoted to the brief consideration of two different subjects, in each of which the experience of Wills' Hospital is certainly large enough to be entitled to respect—the treatment of granular conjunctivitis, and the operation for cataract by division. Of the latter we may say, without hesitation, that our author is, like most Europeans, far behind our native surgeons in experience and success with this operation; and that his account of it is correspondingly unsatisfactory. Dr. Hartshorne, in his note on it, confines himself almost entirely to the opinions and descriptions of Dr. Littell and Dr. Hays, as more authoritative than his own, although he is equally decided in preferring the operation by solution or division to that of reclination, depression or extraction, in the majority of cases. Of the hundreds of operations for cataract performed at Wills' Hospital in the course of twenty-four or five years, a large proportion have been needle operations for solution; and of the whole number, an average of nearly eighty per cent. have recovered tolerable vision. Much of this success may be attributed to the careful treatment of the patient before and after operation, as well as to the nature and mode of the operative procedure. We did not intend, however, to discuss this or any other individual topic, and must pass on to the subject of granulations, with which we must close this already protracted notice.

Granular lids are among the standing pests of all our dispensaries and infirmaries, and notably of our ophthalmic charities. They are brought to us in crowds, even from shipboard, and they swarm from mines and railroad shanties in every quarter. We were certainly not less surprised and disappointed, therefore, than our editor must have previously been, when we found that the whole history of the therapeutic management of this affection was dispatched in twenty-three lines, and that of these, six were spent in unreasonable abuse of "blue stone," and six more in recommending a mode of scarifying the granulated surface, which we consider very bad practice in this region, whatever it may be in London! Dr. Hartshorne's comment extends over about two pages, and although perhaps as much as should be looked for in such a hand-book, and sufficient in the hints it affords for ordinary purposes, it is not a word too full or too long in proportion to the nature of the case discussed.

We subjoin, in conclusion, the author's paragraphs on this vexatious topic, together with the editor's notes thereon in full. The extract may serve as the only specimen we can afford to present of the work of either party.

"*Treatment.*—In the treatment of granular conjunctiva, care and perseverance are required. Carefully conducted diet and regimen, tonics, good air and protection from changes of weather, are important general points of treatment. The local treatment should consist of, 1st, the application of a leech or two to the eyelids [to the temples or behind the ears, or cups to the temples or nuchæ] occasionally to relieve congestion; 2d, counter-irritation, kept up by repeated blisters [or tincture of iodine] behind the ears and over the eyelids; 3d, scarification of the affected conjunctiva (pp. 76, 77) every second or third day, and immediately thereafter the application to it of some strong salve, such as the red precipitate. In scarifying, it is important to lay open the vesicular granulations, one after the other, by puncture. When fungous, the granulations, if large and prominent, and especially if pedunculated, may be at once snipped off with curved scissors. After the operation the salve is to be applied as after the scarification merely. [Occasional scarification to relieve congestion may be very beneficial, like leeching or cupping, in case of plethora or acute relapse; but our experience has satisfied us that, in this country at least, the use of the knife and scissors, as well as of leeches and cups, is often injurious, and that scarification is quite as liable to misuse as the blue-stone.]

"In the treatment of granular conjunctiva, blue-stone, as above observed, (pp. 74, 75,) has been sadly misused. Though by it and other caustics, the granulations may have been destroyed, the conjunctiva has been too often destroyed at the same time. The inflammation, the cure of which ought to be the great object aimed at, has been in general rendered more hopelessly incurable. As to the powdered acetate of lead, I cannot speak of it from much experience.

"[The local remedies which have been found the most useful with us, are the nitrate of silver in solution, (gr. viij. to ℈ij. in the fluid ounce of distilled water, according to the nature of the case,) sulphate of copper in the crystal, and liquor plumbi subacetatis in drops. The first of these, in solutions of various strength, according to the stage and character of the disorder and the susceptibilities of the organ to be acted on, appears to be the most frequently available and beneficial in its operation. It may occasionally be used with advantage, even in the proportion of from three to six grains only to the fluid ounce of water, dropped into the affected eye, once or twice a day, or every two days. Nor can it be tolerated in any other strength, by some patients, especially in the summer season. Generally, however, we prefer resorting to the stronger solutions, (from ten grains to thirty in the fluid ounce,) used at longer intervals and applied with the camel's-hair pencil to the granular surface of the everted lids. This surface should be gently wiped with a soft clean sponge or piece of lint; the pencil, charged with the solution, should then be lightly and rapidly drawn to and fro over the granulations, until they are whitened; after which, the surface thus painted should be once more carefully wiped, and the lid replaced. An application of this kind may be repeated, more or less freely, every two, three, four or six days, according to the amount of irritation and other effects produced.

"The liquor plumbi subacetatis is dropped into the eyes, or on the inner surface of the separated lids, in two or three drops at a time, every two or three days, according to circumstances, in the same manner as the weaker solutions of lunar caustic. Like the stronger solutions of this latter salt it must be used less frequently, and may also be laid on with the brush or pencil in the same way.

"The sulphate of copper is applied, at intervals of two, three or four days, and with the same precautions as in the pencilling with the lunar caustic solutions just referred to. The crayon of "blue-stone" must be smooth on its face, clear and clean, rubbed or filed into a small wedge "of the size and shape of the spade of cards," and fixed in a quill or other convenient holder. It is to be drawn over the parts very lightly, with a single sweep in most instances; although in some indolent cases it may be rubbed on two or three times at one application, with manifest advantage as well as impunity. Sulphate of copper is probably best adapted to the pale form of granulation, in which the reaction, and generally the congestion, are comparatively slight—the subacute or the chronic form; and the mode of using it must depend upon the various and successive grades of irritability, which a cautious and intelligent employment of it may reveal in each case.

"The acute and active, or the congestive, forms of granular conjunctivitis are more likely to be abated by the different strengths of the lunar caustic solution, and by the subacetate of lead. There are, however, so many varieties and shades of irritability, inflammation and granulation between the two extremes of the disease, that the same general rule may serve to regulate the management of all the different remedies; except that of the two last-mentioned, the latter is contra-indicated by the presence of ulceration or any breach of continuity in the parts exposed to its action, and the former, if too long continued, will produce a permanent olive-colored stain. We have, at different times, seen the application of each different agent, in very similar conditions, followed by equally gratifying results, although we are inclined to prefer the lead as a pioneer remedy, in the more acute or inflammatory cases. It is often well to substitute one agent for another; and much good is frequently done by alternating them in the course of the same week; employing, for example, the nitrate of silver, or the blue-stone at discretion, one day, and the subacetate of lead the next, or the second or third day after. When this plan is not adopted, some one of the adjuvants, such as the borax, alum or rock-salt crayon in substance, or solutions of either of these, or the iodide of zinc or the sulphate of zinc and salt solution, or that of tannic acid, or the vinum opii, the diluted citrine ointment or red precipitate ointment being applied to the margins of the lids at night, should be daily employed in support of the strong applications and the general course of treatment. It is obviously impossible, in this volume, to dwell upon contingencies, and to lay down special rules of practice, in the management of this most troublesome and variable affection. Its great prevalence in many parts of the United States, especially among the immigrant population, has induced us to add a few hints to the author's very brief notice. More can be learned from the study of individual cases than from any attempt at detailed directions. For the best American article on the subject, with clinical illustrations, we may refer to the last American edition of Lawrence on the eye, p. 285, et. seq., by Dr. Hays."]

We take pleasure in observing that the book is handsomely printed and amply illustrated. In the latter respect it is particularly well adapted to the wants of a learner, and must prove especially attractive to students. The name of the editor does not appear on the title-page, his share of the responsibility having been assumed at the close of the American advertisement, wherein he very appropriately appends his signature to the only apology which he offers for appearing at all.

*Statistics and Treatment of Typhus and Typhoid Fever, from 12 years experience gained at the Seraphim Hospital in Stockholm,* (1840—1852.) By MAGNUS HUSS, M. D., &c. Translated from the Swedish original, by Ernst Aberg, M. D. London; Longman, Brown, Green & Longman. 1855.

It is well known that there is still a wide diversity of opinion among physicians regarding the identity or non-identity of typhus and typhoid fever; the Irish, the German and the Scandinavian schools regarding them as identical, or nearly related varieties, at most, of the same disease, while the English, the French and the American believe them to be entirely and essentially distinct affections. It is true, that to one not conversant with the literature of the subject, the question of their specific diversity may seem to have been already decided in the affirmative by the investigations and researches of Louis, Gerhard, Jackson, Jenner, Flint and others. An opposite view is held, however, by physicians and pathologists quite as eminent, and whose opinions are, to say the least of them, entitled to respectful consideration. Thus, Dr. Stokes is of opinion that "the advance of knowledge points to the conclusion that, however the continued fevers in the temperate regions of the earth may differ as to certain characters, yet, that they are all closely related; that essentiality appears to be the rule, and its opposite the exception." And Rokitansky, than whom no higher authority can be quoted, when speaking of the "typhus crasis," says, "It comprises the entire nature of the typhous disease, and is at the root of all its phenomena, whether of substantive change or of functional disturbance;" and again, "The typhus crasis manifests a very marked relation to mucous membranes, especially to the lymphatic glands and to the spleen. In middle Europe it is the mucous membrane of the intestine, and especially of the ileum, rarely the bronchial mucous membrane with the lungs and the bronchial glands; in the north, it is rather the last mentioned, namely, the respiratory tract; in the south, (in pest-typhus,) it is the peripheral gland system in which the crasis becomes localized. In the form of a typhous inflammation it determines, in the follicular apparatus of the ileum and in the mesenteric glands, a peculiar marrow-like product, which in intense cases, closely resembles medullary carcinoma."—(Path. Anat. p. 290, Am. Ed.)

In these views the author before us, Dr. Huss, entirely concurs, it being his opinion, drawn from a large experience, that "typhus and typhoid fever, such as they appear in the climate of the north, belong to one and the same pathological process, but that that, which we may call the typhus process, presents several different varieties. These varieties or forms arise from the circumstance that certain groups of symptoms, sometimes from one, sometimes from another of the organs or systems, are more prominent in one individual case than in another. It must certainly be allowed, that the difference may sometimes seem great enough to warrant the admission of distinct diseases, instead of only varieties of the same, but considering the intermediary forms between the two, and how they often change from one to the other and how they unite, the result must be their classification under the same pathological process. It may be very easy to distinguish the two remotest links in the chain, of which the typhus process may be thought to be composed, and then to decide if any particular case is one of typhus or typhoid fever; but even the most penetrating discernment may be baffled in attempting to distinguish one of the intermediary forms, which lie between the two extremes and partake a little of each, and it may perhaps be impossible to make a correct diagnosis."

In his capacity of Physician to the Seraphic Hospital, Dr. Huss enjoyed opportunities of observing and treating these fevers, that seldom fall to the lot of any one individual. During the twelve years from 1840 to 1851 inclusive, the cases received in the hospital under his care and that of his colleague, Prof. Malmsten, numbered 3186. Two severe epidemics, in particular, were studied by him with attention, one of which commenced in September, 1841, and continued till July, 1842; the other in December, 1845 to July, 1846; 503 patients were seen in the first, and 414 during the latter epidemic. "In neither instance were the cases exclusively typhus or typhoid fever—on the contrary, there were some of both; so that in the beginning and to the height of the epidemic, the cases were for the most part typhus, and at the end the typhoid fever almost entirely prevailed. This statement is founded not merely on the symptoms each individual case presented, but also on the results of the post-mortem examinations. With the exception of four, all who died were examined ; there were 55 fatal cases in the former epidemic, 33 in the latter. Of the former 55, 36 presented those alterations of the intestinal tube and mesenterical glands which are peculiar to typhoid fever, and 19 no such alterations. Of the latter 33, only 29 were examined; of these, in 19 the glands were changed in different degrees, the remaining 10 showed no change. During both these epidemics, a contagion or nosocomical miasma developed itself within the hospital, which attacked a few patients who had entered the hospital with other complaints, as well as some of the nurses and medical students. These got what is called nosocomical fever, but the fever agreed in every respect with the prevailing epidemic. Some cases exhibited the petechial form, others the abdominal."

In another more limited epidemic among the gensd'armes, at the barracks, where all the men were between 20 and 40 years of age, lived under the same circumstances, and were exposed to the same influences, the diseases was typhus in some, typhoid in others, and took an intermediate form in the remainder. Of 17 cases also occurring at a cabinet maker's, which Dr. Huss saw within a fortnight, 10 were typhus and 7 typhoid fever.

"The following incident may also merit notice : a man had died, it was stated, of typhus. The brother and his wife went to live in the house of the deceased and used his clothes, without previous airing and cleaning. They were soon taken ill and brought to the hospital, where they both died. The husband had violent delirium and a profuse petechial eruption, the post mortem examination showing no change of the intestinal glands; the wife had milder cerebral symptoms, and a very scarce crop of eruption; but on examination swollen mesenterical glands and swollen and ulcerated peyer plaques were found.

The same experience has been made during those years, when typhus has occurred only sporadic; some cases have taken the characters of typhus, others of typhoid fever, though the latter were, under these circumstances, always more numerous. The season also has in this matter shown a decided influence; sporadic typhus occurring during autumn and winter, while spring and summer have introduced the typhoid cases.

Although these facts are founded on observations made in Stockholm, I believe I may state, with probability if not with absolute certainty, that the same may be said of the rest of Sweden, to judge from the communications received from physicians of different parts of the country."*

Dr. Huss' experience, regarding the symptoms which are especially considered to mark the distinction between typhus and typhoid fever, is "*that the extreme links of the typhus chain, the severer cases of typhus petechialis and the clearly marked ones of typhus abdominalis, are easily distinguished by the absence in the former and presence in the latter, of symptoms, referable to the intestinal tube; but that a number of forms between the two extremes occur, in which this distinction by no means holds good.*"

As regards the results of dissection, he found, "as a rule, the changes of the intestinal and mesenterical glands wanting in the graver forms of typhus petechialis; as exceptions, enlargement sometimes both of the solitary and peyer glands. It has by no means been uncommon though, to see as well enlarged glands as spread ulcerations in milder cases, although with a decided pronounced petechial eruption." "When these enlargements and ulcerations are perfectly similar with those the abdominal form present, it appears, at least to me, very difficult to explain, why the symptoms during life distinctly marking typhus petechialis, it must nevertheless be a case of typhoid fever, only because there have been ulcerated glands. It is true that these ulcerations occur less spread and less in copiousness in the petechial than in the abdominal form, which seems to me to be occasioned *by the existing antagonism between the skin and the intestinal mucous membrane, so that the more copious the eruption is on the skin, the less are the intestinal glands affected, and the contrary.* But we have also cases, with all the symptoms and signs of typhoid fever, the rash included, in which nevertheless the intestinal and mesenterical glands have been healthy. Certainly these cases appertain to rare exceptions, but must not therefore be left unnoticed."

These statements, it must be allowed, are very curious and

* Amongst other information, which has been communicated by my colleagues in the country, the following from *Dr. Fr. Lang* in Gothenbourg, merits special notice, as it proves that the typhus process in other parts of the country agrees, in the respect in question, with that of the metropolis. A traveller came to a small island, situated on the western coast of Sweden. He was sick when he arrived, and was the same day laid up with a fever. The disease showed all the marks peculiar to typhus petechialis, viz. clearly marked alteration of the blood, and a very copious typhus eruption (ecchymotic petechiæ) and ended on the 9th day fatally. Seven persons were successfully taken ill on the island, only one of these presented the marks characterizing typhus; the remaining six cases, of which one was fatal, were all clearly distinct typhoid. The course of this limited epidemic made it evident, that it was produced by infection from the first diseased person who came ill, before whose arrival no case of typhus or typhoid had been seen for several years, either on the island or in the neighborhood, and that the same contagion produced both typhus and typhoid fever.

interesting, and we have no doubt they will be considered as triumphant by all who believe in the identity of the two diseases. There are several circumstances, however, besides their want of agreement with the investigations of other physicians under similar circumstances, which detract considerably from their value. Thus even the author calls the cases where the symptoms alone, unsustained by any changes in the intestinal glands, have diagnosed typhoid fever, 'rare exceptions,'—and we have failed to discover what was their number, whether two, three, five, or a dozen. The same loose way of writing characterizes the whole 'introduction,' the only part of the work in which the subject of the identity of the two diseases is considered, and altogether destroys the scientific value of the author's views. The weight of *exact* testimony in fact is entirely in favor of the non-identity of the two diseases, and we feel sure that Dr. Huss will persuade no one to the contrary who has carefully studied the modern investigations on the subject, upon which none, we take pride in saying, have thrown more light than American physicians.

The remainder of the work is exact and statistical. As the two diseases are united in his tables, however, the author's results lose much value with those who have no confidence in the identity of the two diseases. The work is admirably translated.

---

*Atlas of Cutaneous Diseases.* By J. MOORE NELIGAN, M.D., Edin. M.R.I.A., &c. Philada. Blanchard & Lea. 1856.

The above work, adapted in its references to the author's Treatise on Diseases of the Skin, will be found a very useful assistant by all who wish further information on the affections mentioned than can be obtained by merely verbal descriptions. The English edition is stated to be "cheap in price;" the same statement may be safely made, we have no doubt, regarding the American reprint. The work contains sixteen plates and ninety figures.

---

*On some of the Diseases of Women admitting of Surgical Treatment.* By ISAAC BAKER BROWN, F.R.C.S. (By Exam.) &c. Illustrated by twenty-four Engravings. Philada.; Blanchard & Lea. 1856.

Our limited space in this number precludes the possibility of any extended comments upon the above work at the present time. We have carefully examined its contents, and have no hesitation in recommending it as the production of a surgeon who has had much experience in the subjects it discusses. Dr. Marion Sims' treatment of vesico-vaginal fistula is, we are glad to find, fully described. The suture used by Dr. Sims, the author believes to be the best now known.

